# The causal relationship between hypothyroidism and frozen shoulder: A two-sample Mendelian randomization

**DOI:** 10.1097/MD.0000000000035650

**Published:** 2023-10-27

**Authors:** Guanghua Deng, Yongkang Wei

**Affiliations:** a Ya’an Hospital of Traditional Chinese Medicine, Sichuan, China; b The Fourth Clinical College of Xinjiang Medical University, Xinjiang, China.

**Keywords:** frozen shoulder, hypothyroidism, Mendelian randomization

## Abstract

To investigate the causal relationship between hypothyroidism and frozen shoulder using a Mendelian randomization (MR) approach. Pooled data from a large-scale genome-wide association study (GWAS) were used. Genetic loci that were independent of each other and associated with hypothyroidism and frozen shoulder in populations of European ancestry were selected as instrumental variables. Inverse variance weighting (IVW) was used as the primary analysis method. Weighted median (WME) and MR-Egger were used as complementary analysis methods to assess causal effects. To explore the causal relationship between hypothyroidism and frozen shoulder. Sensitivity test analysis was performed using heterogeneity test, multiple validity test, and leave-one-out analysis to explore the robustness of the results. IVW results showed an OR (95% CI) of 1.07 (1.01–1.14), *P* = .024, indicating that hypothyroidism is a risk factor for a frozen shoulder. And no pleiotropy was found by the test, and sensitivity analysis also showed robust results. This study used 2-sample MR analysis to analyze and explore the genetic data, and the results showed a higher prevalence of frozen shoulder in patients with hypothyroidism, suggesting that active control of hypothyroidism may reduce the occurrence of frozen shoulder.

## 1. Introduction

Frozen shoulder, also known as adhesive capsulitis,^[1]^ is a pathological condition characterized by pain and limited joint motion in the shoulder.^[2]^ There are usually no significant findings in the patient history, clinical examination, or imaging evaluation to explain the loss of motion or pain.^[3,4]^ Hypothyroidism is a common disorder characterized by an insufficient amount of thyroid hormones provided by the thyroid gland to meet the needs of peripheral tissues.^[5]^ Hypothyroidism can cause several complications,^[6–8]^ and retrospective studies have been conducted to suggest that hypothyroidism increases the incidence of frozen shoulder.^[9]^ However, the association between hypothyroidism and frozen shoulder may be influenced to some extent by the confounding factors and reverse causality inherent in traditional observational studies.^[10]^ Therefore, the causal relationship between hypothyroidism and frozen shoulder still needs further investigation.

Mendelian randomization (MR), a genetic epidemiological method, is a useful tool to assess the causal role of hypothyroidism and frozen shoulder.^[11]^ By using genetic variants such as single nucleotide polymorphism (SNP) as instrumental variables that can modify disease risk factors or exposures. MR studies can strengthen the causal inference of exposure-outcome associations.^[12]^ According to Mendel law of inheritance, genetic variants are not susceptible to confounding factors because they are randomly assigned during gamete formation.^[13]^ In addition, confounding factors and reverse causality can be minimized because genotypes cannot change with disease progression.^[14]^

To this end, we conducted a 2-sample MR study to examine the association of genetic susceptibility to hypothyroidism with frozen shoulder risk factors. We aimed to provide important evidence for the causal role of hypothyroidism in causing a frozen shoulder.

## 2. Data and Methods

### 2.1. Data sources

The largest sample size of genome-wide association study (GWAS) data for hypothyroidism and frozen shoulder was obtained through the IEU OpenGWAS project (mr cieu. ac. uk). The website was accessed on 2023-06-06. The final population source for all data was Europe, of either sex. This includes hypothyroidism (finn-b-HYPOTHYROIDISM) containing 16,378,441 SNPs, 26,342 in the observation group and 59,827 in the control group, and frozen shoulder (ebi-a-GCST90000512) containing 15,184,371 SNPs with a sample size of 451,099. This study was a re-analysis of previously collected and published data and therefore did not require additional ethical approval.

### 2.2. Conditions for SNPs as instrumental variables

First, the instrumental variable was highly correlated with exposure, with F > 10 as a strong correlation criterion.^[15]^ Secondly, the instrumental variable was not directly correlated with the outcome, but only influenced the outcome through exposure, meaning that there was no genetic pleiotropy. In this study, the MR-Egger regression model with a non-zero intercept term (*P* < .05) indicated the absence of genetic pleiotropy.^[16]^ Third, instrumental variables were not related to unmeasured confounding.^[17]^ The human genotype-phenotype association database Phenoscanner V2 was searched for associated phenotypes of instrumental variables at genome-wide significance levels to determine whether these SNPs were associated with potential risk factors.^[18]^

### 2.3. SNP screening

Significant SNPs were screened from the GWAS pooled data of hypothyroidism (with *P* < 5 × 10- 8 as the screening condition)^[19]^; the linkage disequilibrium coefficient r^2^ was set at 0.001 and the width of the linkage disequilibrium region was 10000 kb to ensure that individual SNPs were independent of each other^.[20]^ The above-screened hypothyroidism-related SNPs were extracted from the GWAS summary data of frozen shoulder, while SNPs directly related to outcome indicators (*P* < 5 × 10- 8) were excluded. The F value of each SNP was calculated, and SNPs with weak instrumental variables (F value < 10) were excluded.^[21]^ And the human genotype-phenotype association database was queried to screen for potentially relevant risk factor SNPs and to exclude them.^[22]^

### 2.4. Causality validation methods

The causal relationship between exposure (hypothyroidism) and outcome (frozen shoulder) was verified mainly using inverse variance weighting (IVW), supplemented by weighted median (WME) and MR-Egger MR analysis methods, using SNPs as instrumental variables.

### 2.5. Sensitivity analysis

Various methods were used for sensitivity analysis. First, the Cochran Q test was used to assess the heterogeneity among the individual SNP estimates, and a statistically significant Cochran Q test proved that the analysis was significantly heterogeneous. Second, the MR pleiotropy residual sum and outlier (MRPRESSO) was used to validate the results in the IVW model, correct for the effects of outliers, and if outliers existed, they were excluded and the analysis was repeated. Third, the MR-Egger intercept test was used to test the horizontal multiplicity of SNPs. If the intercept term in the MR-Egger intercept test analysis was statistically significant, it indicated that the MR analysis had significant horizontal multiplicity. Fourth, leave-one-out analyses were performed by removing a single SNP at a time to assess whether the variation drove the association between the exposure and outcome variables. Fifth, funnel plots and forest plots were constructed to visualize the results of sensitivity analyses. *P* < .05 suggests a potential causal relationship for MR analysis and is statistically significant. All statistical analyses were performed using the “TwoSampleMR” package in R software version 4.3.0.

## 3. Results

### 3.1. Instrumental variables

The current study screened 32 SNPs that were strongly associated with hypothyroidism (*P* < 5 × 10- 8) without chain imbalance (r^2^ < 0.001, kb = 10,000). 29 SNPs remained by taking intersection with SNPs in the GWAS pooled data from frozen shoulder and also excluding SNPs directly associated with outcome indicators. In our study, each SNP had an F value >10, indicating no weak instrumental variables (Table 1). We searched the human genotype-phenotype association database and no potentially relevant risk factor SNPs were found.

**Table 1:** Information on the final screening of hypothyroidism SNPs from GWAS data (n = 29).

ID	SNP	Effect_Allele	Other_Allele	β	SE	P值	F值
1	rs10760344	T	G	0.1034	0.014718	4.96E-13	52
2	rs10974437	G	A	−0.1028	0.015635	1.72E-08	31
3	rs10983700	C	T	0.2137	0.014747	5.18E-50	220
4	rs11171710	A	G	−0.0764	0.014061	2.97E-08	30
5	rs1203943	C	T	0.0947	0.016899	1.81E-08	31
6	rs1317983	C	T	0.1137	0.015048	1.12E-14	59
7	rs143117642	A	G	−0.2064	0.047181	3.68E-09	34
8	rs1534430	T	C	−0.0842	0.014255	8.64E-10	37
9	rs17008423	T	C	−0.1186	0.01729	2.33E-08	31
10	rs17364832	G	T	0.0909	0.015499	1.06E-09	37
11	rs17786733	A	T	0.0785	0.014195	1.18E-08	32
12	rs1915930	T	G	0.0954	0.013939	1.03E-11	46
13	rs1993945	T	A	0.1258	0.014293	5.98E-20	83
14	rs2110451	A	G	0.0906	0.015205	2.04E-09	36
15	rs2111485	G	A	0.0799	0.014213	7.35E-09	33
16	rs229531	C	T	0.0826	0.014034	2.38E-09	35
17	rs2553610	G	C	−0.0778	0.014175	1.03E-08	32
18	rs2844542	G	C	0.1087	0.0141	2.28E-13	53
19	rs3008034	C	T	−0.0879	0.015079	1.19E-08	32
20	rs3087243	A	G	−0.1302	0.013951	3.11E-19	80
21	rs4274624	T	C	−0.1121	0.016639	2.59E-12	49
22	rs597808	G	A	−0.1638	0.013923	8.70E-33	142
23	rs6679677	A	C	0.3052	0.022922	5.47E-60	266
24	rs7599564	G	A	0.0792	0.014264	1.80E-08	31
25	rs7754251	C	G	0.0819	0.014063	1.82E-09	36
26	rs897586	A	G	−0.0805	0.014502	3.83E-08	30
27	rs9271365	G	T	0.2357	0.014044	3.40E-56	250
28	rs9277457	G	C	−0.1179	0.015063	9.50E-12	46
29	rs9497965	T	C	0.0935	0.014154	8.44E-11	42

SNP = single nucleotide polymorphism.

### 3.2. Causal relationship between Hypothyroidism and frozen shoulder

**Figure 1.**
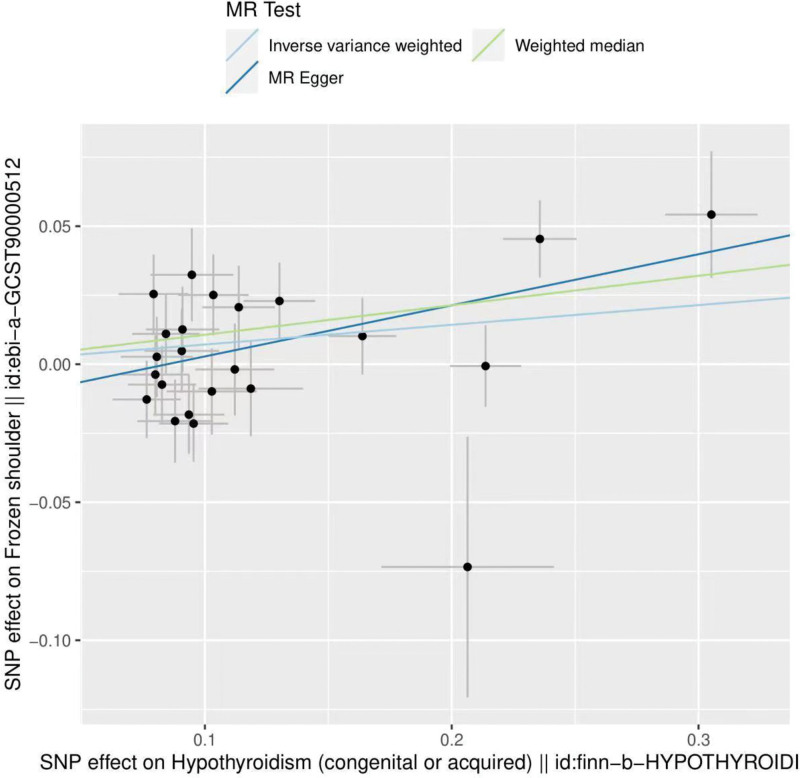
Scatter plot of hypothyroidism and frozen shoulder.

**Figure 2.**
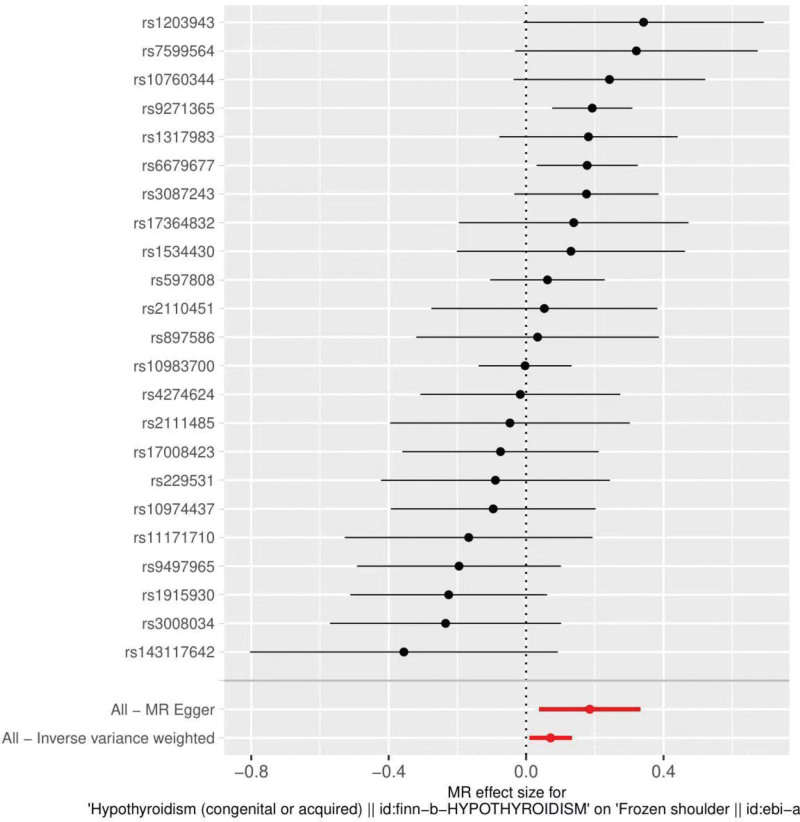
Forest plot of hypothyroidism and frozen shoulder.

By MR analysis, the results of IVW, WME, and MR-Egger showed a causal relationship between hypothyroidism and frozen shoulder. IVW: OR = 1.07, 95% CI = 1.01–1.14, *P* = .024; WME:OR = 1.11, 95% CI = 1.03–1.20, *P* = .008; MR-Egger: OR = 1.20, 95% CI = 1.04–1.40, *P* = .023 (Table 2). We can see from both the scatter plot (Fig. 1) and the forest plot (Fig. 2) that hypothyroidism increases the risk of developing a frozen shoulder.

**Table 2:** MR regression results of the 3 methods.

Method	β	SE	OR (95% CI)	*P*
IVW	0.071	0.032	1.07 (1.01–1.14)	.024
WME	0.107	0.041	1.11 (1.03–1.20)	.008
MR-Egger	0.185	0.075	1.20 (1.04–1.10)	.023

IVW = inverse variance weighting, WME = weighted median.

### 3.3. Sensitivity analysis

**Figure 3.**
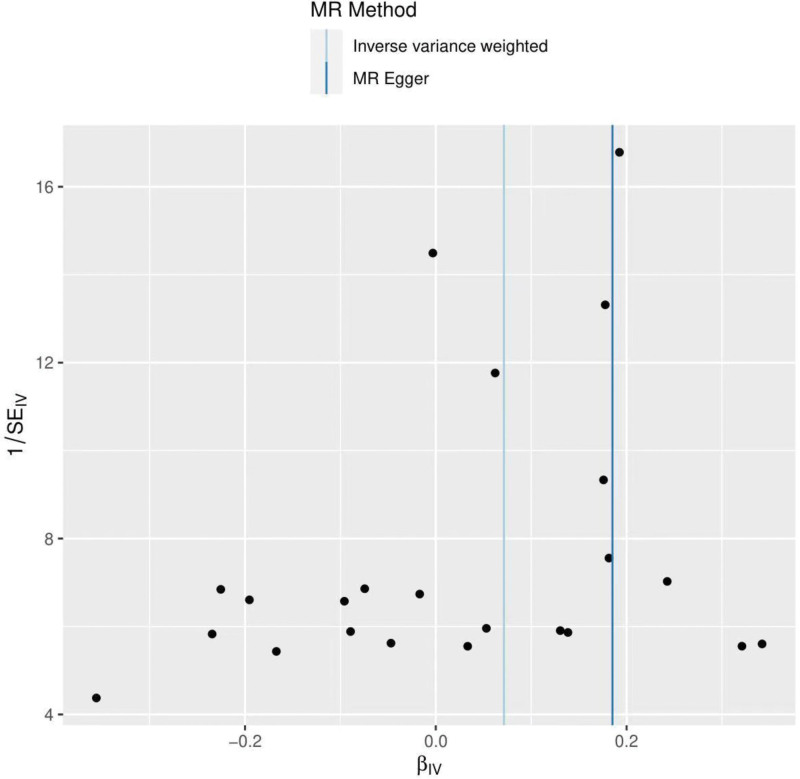
Funnel plot of hypothyroidism and frozen shoulder.

**Figure 4.**
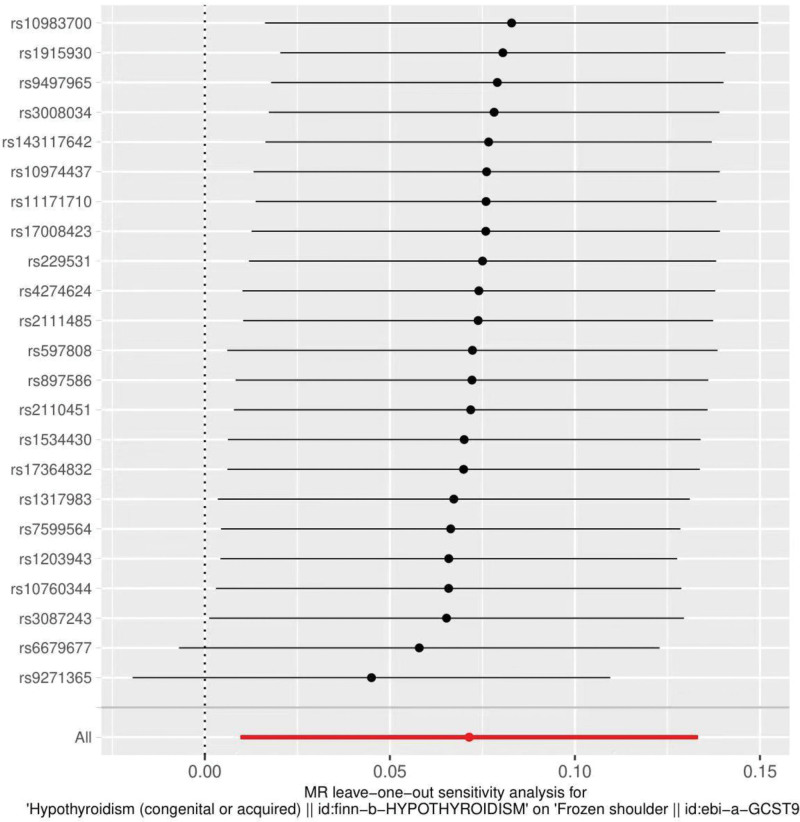
Analysis of hypothyroidism and frozen shoulder by the leave-one-out method.

The heterogeneity test was performed using MR Egger and IVW methods (MR Egger *P* value = 0.08; IVW *P* value = 0.04), MR Egger method suggested no heterogeneity, but IVW results suggested heterogeneity. Funnel plots were drawn to show the heterogeneity results, as shown in Figure 3. Using MR-PRESSO for screening SNPs that may cause heterogeneity, no SNPs were found to cause heterogeneity in the results, and at this point, we should focus on the results of the IVW random effects model. The results of the global test by MR-PRESSO suggested no pleiotropy (*P* = .11). The IVW method was used by default for the leave-one-out method, and as seen in Figure 4, the results for the remaining SNPs after excluding any of them are on the right side of the valid line, indicating that the results are robust.

## 4. Discussion

Hypothyroidism is known to be a possible risk factor for a frozen shoulder, but the causality of this association is unclear. Our MR study aimed to reveal the causal relationship between hypothyroidism and a frozen shoulder. The results of the 2-sample MR showed that hypothyroidism was a risk factor for a frozen shoulder with an OR (95% CI) of 1.07 (1.01–1.14). It indicates that the risk of developing a frozen shoulder is 1.07 times higher in patients with hyperthyroidism compared to the general population.

A study by Milgrom et al,^[23]^ after analyzing 126 patients with frozen shoulders and 98 control cases in the city of Jerusalem, found that thyroid disease was not only a risk factor for musculoskeletal disorders in general, but also a specific risk factor for frozen shoulders in women. Cakir et al study^[24]^ found that frozen shoulder was present in 10.9% of cases in patients with thyroid disease, with a significantly higher prevalence than in the general population. In addition, a study by Schiefer et al^[9]^ found a significantly higher prevalence of hypothyroidism diagnosis in the group with frozen shoulder (27.2% vs 10.7%; *P* = .001) after comparing 93 patients with frozen shoulder to 151 control cases. And there was also a trend toward a higher prevalence of bilateral frozen shoulder in patients with elevated TSH levels (*P* = .09). Finally, a study by Park et al^[25]^ found that hypothyroidism was significantly associated with a frozen shoulder (OR:2.10; 95% CI:1.36–3.15; *P* = .001) after comparing 412 patients with frozen shoulder and 1236 normal individuals without frozen shoulder.

This study confirms the risk factors for hypothyroidism and frozen shoulder from a genetic perspective. Although how hypothyroidism plays this role is unclear and further in-depth studies are needed.

The results of the current study are consistent with previous findings that hypothyroidism is a risk factor for the development of a frozen shoulder. People with hypothyroidism are more likely to develop frozen shoulders. Therefore, patients with hypothyroidism should be treated promptly with thyroid hormone supplementation. And screening for frozen shoulders in hypothyroidism should be increased to detect patients with a frozen shoulder at an early stage and treat them in time, which is beneficial to their prognosis.

At the same time, there are some limitations to this study. First, because all data were from a population of European ancestry, the results do not represent a truly randomized population sample and do not apply to other so races. Second, although various sensitivity analyses have been performed in this study to test the hypotheses of the MR study, it is also difficult to completely rule out horizontal pleiotropy of instrumental variables. Finally, the current sample size of GWAS data is still not large enough, and more in-depth studies using more GWAS data are needed in the future.

## 5. Conclusion

In conclusion, this study used a 2-sample MR analysis to analyze and explore the genetic data, and the results showed a higher prevalence of frozen shoulder in hypothyroid patients, suggesting that active control of hypothyroidism may reduce the occurrence of frozen shoulder.

## Author contributions

**Conceptualization:** Yongkang Wei.

**Data curation:** Guanghua Deng.

**Formal analysis:** Guanghua Deng.

**Investigation:** Guanghua Deng.

**Methodology:** Guanghua Deng.

**Project administration:** Guanghua Deng.

**Resources:** Guanghua Deng.

**Software:** Guanghua Deng.

**Supervision:** Guanghua Deng.

**Validation:** Guanghua Deng.

**Visualization:** Guanghua Deng.

**Writing – original draft:** Guanghua Deng.

**Writing – review & editing:** Yongkang Wei.
